# Sequence Features Contributing to Chromosomal Rearrangements in *Neisseria gonorrhoeae*

**DOI:** 10.1371/journal.pone.0046023

**Published:** 2012-09-24

**Authors:** Russell Spencer-Smith, Eldho M. Varkey, Mark D. Fielder, Lori A. S. Snyder

**Affiliations:** Kingston University, School of Life Sciences, Kingston upon Thames, United Kingdom; The University of Hong Kong, Hong Kong

## Abstract

Through whole genome sequence alignments, breakpoints in chromosomal synteny can be identified and the sequence features associated with these determined. Alignments of the genome sequences of *Neisseria gonorrhoeae* strain FA1090, *N.* *gonorrhoeae* strain NCCP11945, and *N. gonorrhoeae* strain TCDC-NG08107 reveal chromosomal rearrangements that have occurred. Based on these alignments and dot plot pair-wise comparisons, the overall chromosomal arrangement of strain NCCP11945 and TCDC-NG08107 are very similar, with no large inversions or translocations. The insertion of the Gonococcal Genetic Island in strain NCCP11945 is the most prominent distinguishing feature differentiating these strains. When strain NCCP11945 is compared to strain FA1090, however, 14 breakpoints in chromosomal synteny are identified between these gonococcal strains. The majority of these, 11 of 14, are associated with a prophage, IS elements, or IS-like repeat enclosed elements which appear to have played a role in the rearrangements observed. Additional rearrangements of small regions of the genome are associated with pilin genes. Evidence presented here suggests that the rearrangements of blocks of sequence are mediated by activation of prophage and associated IS elements and reintegration elsewhere in the genome or by homologous recombination between IS-like elements that have generated inversions.

## Introduction

Genomic rearrangements in pathogenic *Neisseria* species have been observed on three evolutionary levels, within species, strains, and strain variants. Macrorestriction maps of *Neisseria meningitidis* strain Z2491 and *Neisseria gonorrhoeae* strain FA1090, have shown not only a strong inter-species relationship, but also widespread chromosomal rearrangements between the species [1].

These bacterial species contain an extensive array of repetitive sequences that play a number of roles in the biology of the organism. The *Neisseria* spp. phase vary expression of several surface structures through changes in simple sequence repeats [2], contributing to antigenic variation, immune evasion, and niche adaptation [3]–[5]. Tandem repeats can both mediate phase variation [6] and alter the protein sequence [7]. Families of IS elements and other elements, such as DNA uptake sequence (DUS) and Correia Repeat Enclosed Elements (CREE) [8], [9], are repeated throughout the genome [10]. Such repeated units are homologous and therefore are targets for recombination, which may result in the generation of duplications, deletions, rearrangements, and general genomic plasticity [11].

In *N. meningitidis*, the comparative analysis of three meningococcal genomes by Bentley *et al*., [12] revealed that at each of the three major inversion event (IE) locations, repeats are involved. IE1 appears to have arisen due to an inversion between repeat arrays in *N. meningitidis* strain Z2491. In meningococcal strain FAM18, IE2 is believed to be the result of recombination between copies of IS1106. Lastly, IE3 in *N. meningitidis* strain MC58 is associated with paralogous copies of genes that may have been substrates of recombination [12]. More recently, analyses of 20 genome sequences has attributed rearrangements in *N. meningitidis* to dRS3 repeats and IS elements [13]. Species specific features, including IS elements [14] and prophage [10], differentiate the meningococcus from the rest of the genus. In addition, when comparing *N. meningitidis* strain MC58 and *N. gonorrhoeae* strain FA1090, there are fewer dRS3 repeat elements (689 vs 208), IS elements (29 vs 16), and complete Correia Repeat elements (251 vs 122) in *N. gonorrhoeae* [15]. It is therefore possible that rearrangements within *N. gonorrhoeae* are mediated by different mechanisms from those proposed for *N. meningitidis* [13].

Previous comparisons against *N. meningitidis* genome sequences showed rearrangement in FA1090 relative to the meningococci [10]. Since that time, other *N. gonorrhoeae* genome sequences have been completed [16], [17]. Alignment of the two genome sequences of *N. gonorrhoeae* strains FA1090 and NCCP11945 revealed that there has been extensive chromosomal rearrangement between the two strains of this species [18]. Four significant sized inversions were noted, along with a multitude of rearrangements encompassing much of the genome, although only the inversion mediated by Correia Repeat Enclosed Elements (CREE) has been investigated previously in *N. gonorrhoeae* [18].

In addition, rearrangements in strain variants have been reported. A large inversion of more than a third of the gonococcal chromosome was seen between pilin variants N137 and N138, of *N. gonorrhoeae* strain MS11 [19]. Other variants of this strain also exhibited amplification of a 26 kb region that is present in a single copy, in duplicate, or in triplicate [19]. This work, from 1996, was the first demonstration of major chromosomal rearrangements in this species.

Several kinds of repeats have been suggested to play a role in neisserial chromosomal rearrangements. Correia Repeat Enclosed Elements (CREE) are present over 100 times in the neisserial genome and appear to be hotspots for recombination and rearrangements [9], [18], [20], [21]. Comparative evidence supports the involvement of these elements in chromosomal rearrangement in *N. gonorrhoeae* [18].

Evidence shows IS1016 plays a crucial role in genome-wide rearrangements seen between *N. gonorrhoeae* strain FA1090 and in *N. meningitidis* strain Z2491 [22]. There is also evidence that ISNgo2 has a role in large scale genome rearrangements [23]. It has been hypothesized that ISNgo elements may play a role in filamentous phage insertion into the *N. gonorrhoeae* genome [24].

Previous *in silico* analysis of *N. gonorrhoeae* strain FA1090 has revealed the presence of nine integrated prophage, some of which are complete and potentially active [24], [25]. Significant regions of homology are seen between dsDNA phage NgoΦ1, NgoΦ2 and NgoΦ3. NgoΦ1 and NgoΦ2 are thought to be fully functioning phage, as analysis shows all the genes required for lytic growth, while NgoΦ3, NgoΦ4, and NgoΦ5 are incomplete. All four ssDNA phage (NgoΦ6, NgoΦ7, NgoΦ8, and NgoΦ9) show high similarity to each other and are associated with ISNgo2 and ISNgo3 IS-like elements. The phage are therefore thought to integrate into the genome via their own transposase, known as the phage-as-integrase model [24]. Although the ssDNA phage share similarity to those in *N. meningitidis*, the dsDNA phage are not found in the meningococcus [25] and the meningococcal Mu-like phage [26] and phage PNM1 [27] are not see in *N. gonorrhoeae*.

The availability of a third complete *N. gonorrhoeae* genome sequence makes meaningful analyses of the chromosomal rearrangements in the gonococcus possible. *N. gonorrhoeae* strain FA1090 (Accession AE004969) was isolated from an American patient in 1981 [28]. Strain NCCP11945 was isolated from a Korean patient in 2002 [17] and strain TCDC-NG08107 was isolated from a Taiwanese patient in 2008 [16]. Using the genome sequence data from these strains, the implications of the locations of CREE, IS elements, and prophage have been explored in light of chromosomal rearrangements that can be observed.

## Materials and Methods

### Alignment of Genome Sequence Data

**Figure 1:**
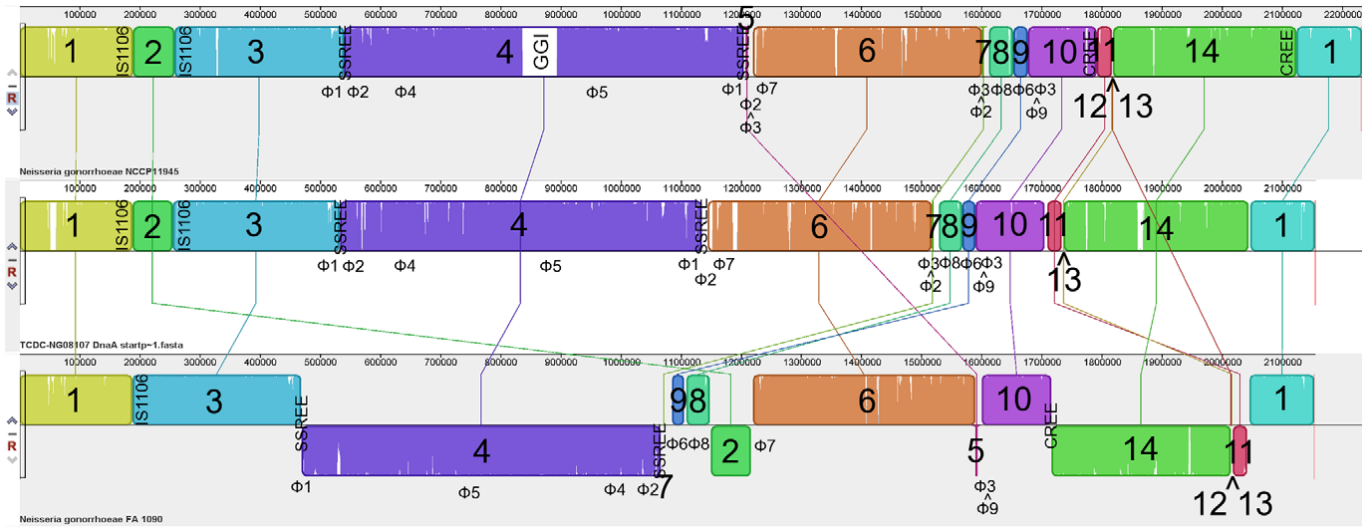
Three-way Mauve alignment of *N. gonorrhoeae* strains NCCP11945 (top), TCDC-NG08107 (middle), and FA1090 (bottom). The numbered blocks represent regions of homology between strains as determined by progressive Mauve alignment on default settings. White vertical lines within blocks represent small localized areas of the genome sequences that have not aligned. The largest of these is the Gonococcal Genetic Island (GGI), which is present in strain NCCP11945 but not the other two strains. Blocks below the central line represent sequences that are inverted in comparison to the strain NCCP11945 arrangement. Homologous blocks are numbered 1 to 14. Blocks 5 and 12 are absent from TCDC-NG08107. Positions of prophage sequences, some of which are fragmented, are indicted: NGOФ1 (Ф1); NGOФ2 (Ф2); NGOФ3 (Ф3); NGOФ4 (Ф4); NGOФ5 (Ф5); NGOФ6 (Ф6); NGOФ7 (Ф7); NGOФ8 (Ф8); and NGOФ9 (Ф9). Labeled are the positions of block 2 flanking IS1106 elements, the block 4 flanking SSREE elements, and the block 11–14 flanking CREE in strain NCCP11945. Note that the chromosomal positions for strain TCDC-NG08107 do not equate to those in GenBank; position 1 was adjusted to be the first base of *dnaA* to facilitate comparisons.

**Figure 2:**
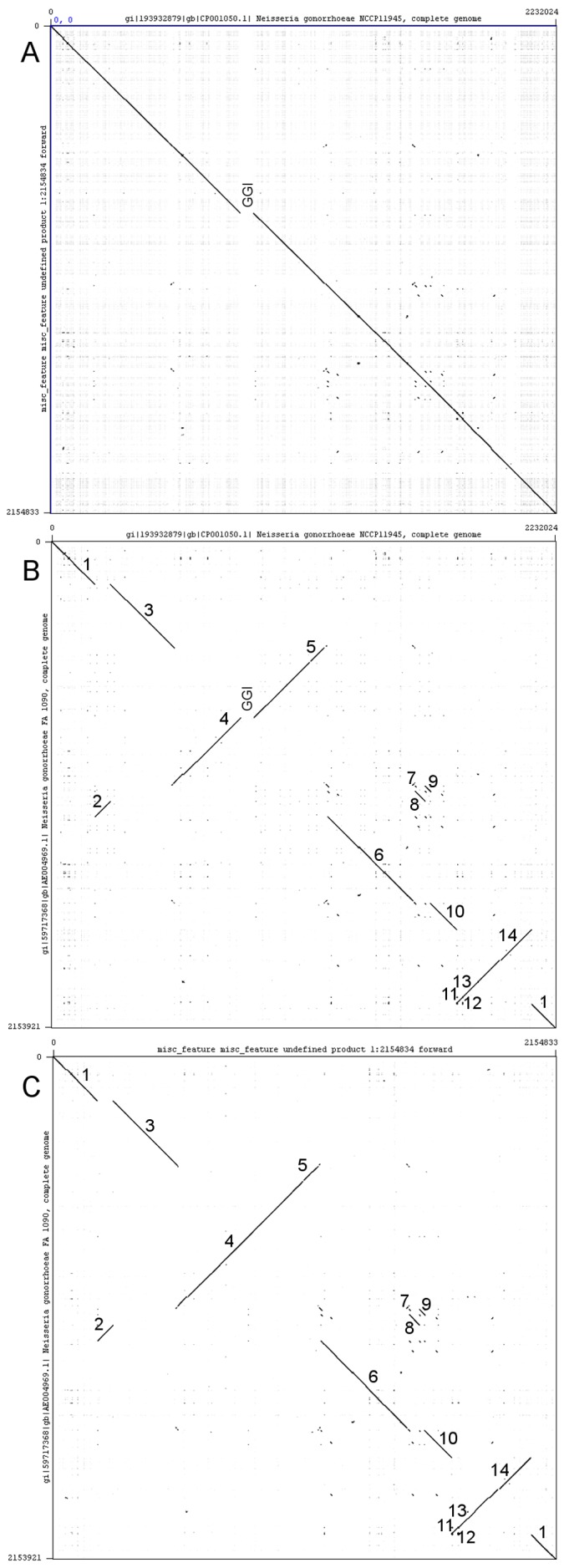
Dot plot alignments of the genome sequences of *N.* *gonorrhoeae* strains NCCP11945, TCDC-NG08107, and FA1090. Panel A: alignment of the complete genome sequence of strain NCCP11945 (horizontal) against TCDC-NG08107 (vertical); Panel B: alignment of the complete genome sequence of strain NCCP11945 (horizontal) against FA1090 (vertical); Panel C: alignment of the complete genome sequence of strain TCDC-NG08107 (horizontal) against FA1090 (vertical). The Gonococcal Genetic Island (GGI), present in strain NCCP11945, is indicated in panels A and B. For panels B and C, block numbers from Figure 1 are indicated. Reverse slopes indicate inversions of sequence between the two genomes being compared.

The genome sequences of *N. gonorrhoeae* strains FA1090 (AE004969), NCCP11945 (CP001050), and TCDC-NG08107 (CP002440) were aligned against one another using progressive Mauve v2.3.1 on default settings [29]. These results are in Figure 1. Additionally, dot plots were generated from each pair-wise comparison of the genome sequence data (Figure 2) using Gepard v1.3 on default settings [30]. To facilitate the interpretation of the results of these alignments, the strain TCDC-NG08107 sequence data (CP002440), was adjusted so that the first base of *dnaA* was at the start of the sequence file, as it is for strains FA1090 and NCCP11945.

Genomic synteny was also assessed using WebACT (available from http://www.webact.org/). WebACT was designed and built by James Abbott (Centre for Bioinformatics, Imperial College, London) and David Aanensen (Department of Infectious Disease Epidemiology, Imperial College London) in the laboratory of Prof. Brian Spratt with much appreciated assistance from Kim Rutherford (FlyMine, Department of Genetics, University of Cambridge).

### Locating and Analyzing Insertion Sequences

The sequences of the IS elements were obtained from ISFinder (http://www-is.biotoul.fr/) [31] and used with MegaBLAST from the NCBI to identify all of the IS elements in *N. gonorrhoeae* strains FA1090 (AE004969), NCCP11945 (CP001050), and TCDC-NG08107 (CP002440). The results were tabulated and compared (Tables S1, S2, S3). Variations where noted between the annotations of the regions identified, both in terms of nomenclature and length of sequence feature. Relative IS element locations were compared to each other, to chromosomal breakpoints, and to prophage locations throughout the three sequences using a three-way progressive Mauve alignment v. 2.3.1 [29]. Sequence homology and annotations were compared in each instance to determine IS element translocations. Prophage and other IS elements 5′ and 3′ of the IS elements along with genes within the central region were analyzed to identify potential composite transposon activity.

### Locating Prophage Gene Clusters

Using Artemis [32], the prophage sequences within the *N.* *gonorrhoeae* strain FA1090 genome sequence were excised in FASTA format and NCBI Mega-BLAST [33] was employed to search for similarity within *N. gonorrhoeae* strains NCCP11945 and TCDC-NG08107; hits were recorded. Additional prophage sequences that might not be present in strain FA1090 were sought using prophage sequences from other *Neisseria* spp., other bacteria, and through analysis of the genome sequence annotations. Searches of Genbank and the research literature revealed previously identified prophages of the *Neisseria* spp. and other bacterial species. In addition, annotations for ‘prophage associated genes’ and genes commonly found in prophage genomes were assessed. The resulting sections of homology were then manually pieced together to give prophage gene clusters throughout the genome (Tables S4, S5, S6, S8, S9, S10, S11, S12, S13). The fully annotated Genbank sequences of *N. gonorrhoeae* strains NCCP11945 and TCDC-NG08107 were aligned pair-wise against *N. gonorrhoeae* strain FA1090 using Progressive Mauve. The prophage gene clusters were aligned gene by gene using strain FA1090 as a template and “best matches” were recorded. The breakpoints and flanking regions for prophage gene clusters that appeared to have changed loci were analyzed using NCBI’s ORF finder (Tatusov & Tatusov, http://www.ncbi.nlm.nih.gov/projects/gorf/), EMBOSS einverted inverted repeat finder [34], EMBOSS Fuzznuc, and Artemis to confirm changes had occurred as well as to locate genes/elements that may be the cause of these translocations. The origins of regions within prophage gene clusters were analyzed using MBCF oligo calculator (http://mbcf.dfci.harvard.edu/docs/oligocalc.html) for GC content, which was then be compared to the already defined GC content of the prophage of strain FA1090 [24], [25]. Finally, the relative locations of the putative prophage were mapped against those of *N.* *gonorrhoeae* strain FA1090 using Microsoft Visio to create an overview of possible chromosomal/prophage rearrangement due to prophage activity.

## Results

### Chromosomal Rearrangements

Overall the configuration of *N. gonorrhoeae* strains NCCP11945 and TCDC-NG08107 are very similar, as seen in both the Mauve alignment and dot plot of the two strains (Figures 1 and 2A). NCCP11945 contains the Gonococcal Genetic Island [18]; this is the largest variation seen between these two strains.

In contrast, large differences in chromosomal arrangement are seen between *N. gonorrhoeae* strain FA1090 and NCCP11945/TCDC-NG08107, where large segments of the DNA sequence have been reordered. Dot plot comparisons of strain FA1090 with strain NCCP1145 and of strain FA1090 with TCDC-NG08107 indicate inversions and translocations of sequence (Figure 2B and 2C). Mauve alignments of gonococcal strains NCCP11945, TCDC-NG08107, and FA1090 (Figure 1) shows a total of 14, 12, and 14 blocks of similarity, flanked by 14, 12, and 14 breakpoints, respectively (Table 1). These reflect those seen on dot plots (Figure 2). Two small sections, represented as blocks 5 and 12 in Figure 1, are missing from *N. gonorrhoeae* strain TCDC-NG08107. Blocks 2, 4, 5, 7, 11, 12, 13, and 14 in Figure 1 are found to be inverted relative to *N. gonorrhoeae* strain NCCP11945 and are thus depicted as below the central line in strain FA1090 in the Mauve figure (Figure 1) and as reversed slopes on dot plot (Figure 2B). As well as being inverted, block 2 is found to have been transposed by almost 1 Mb when the three gonococcal strains are compared. Blocks 7, 8, and 9 have also translocated both across the genome and relative to one another (Figure 1), with block 7 inverted in strain FA1090 and followed by block 9.

**Table 1:** Blocks of homologous sequence in *N. gonorrhoeae* genome sequences.

Block*	NCCP11945∧	TCDC-NG08107†	FA1090‡	Notes
1	2,123,420–189,030	2,033,007–174,421	2,046,256–187,540	
2	189,287–257,387	174,678–241,363	1,149,213–1,216,947	Inverted; flanked by IS1106 and containing an ISNgo2 element
3	257,388–540,606	241,364–518,860	187,542–468,949	
4	540,716–1,205,338	519,705–1,120,703	468,950–1,065,827	Inverted; between SSREE1 of NGOΦ1 and SSREE2 of NGOΦ2
5	1,207,883–1,211,731	not present	1,590,108–1,593,758	Inverted; associated with NGOΦ3
6	1,219,580–1,600,160	1,131,043–1,502,991	1,219,107–1,590,089	
7	1,600,161–1,604,818	1,502,992–1,507,692	1,070,406–1,072,535	Associated with NGOΦ2
8	1,612,648–1,652,746	1,515,175–1,554,400	1,109,820–1,148,016	NGOΦ8 with genomic sequence; flanked by ISNgo2 elements
9	1,652,992–1,676,401	1,554,453–1,576,630	1,084,792–1,105,090	NGOΦ6 with rRNA locus; flanked by ISNgo2 elements
10	1,676,402–1,791,478	1,576,631–1,691,738	1,600,924–1,716,026	
11	1,791,637–1,816,795	1,696,304–1,720,643	2,016,883–2,042,146	Inverted with 12, 13, and 14; associated with CREE (Snyder *et al*., 2009)
12	1,816,891–1,817,595	not present	2,014,348–2,015,347	Inverted with 11, 13, and 14
13	1,818,227–1,818,872	1,723,126–1,723,210	2,015,535–2,016,612	Inverted with 11, 12, and 14
14	1,819,903–2,123,077	1,723,233–2,031,018	1,716,027–2,014,266	Inverted with 11, 12, and 13; associated with CREE (Snyder *et al*., 2009)

* Block numbers are from Mauve genome alignments shown in Figure 1.

∧ Genomic positions from *N. gonorrhoeae* strain NCCP11945 (CP001050).

† Genomic positions from *N. gonorrhoeae* strain TCDC-NG08107 (CP002440).

‡ Genomic positions from N. gonorrhoeae strain FA1090 (AE004969).

### A Large Inversion Affecting the Lysogenic dsDNA Prophage and the Spencer-Smith Repeat Enclosed Element

**Figure 3:**
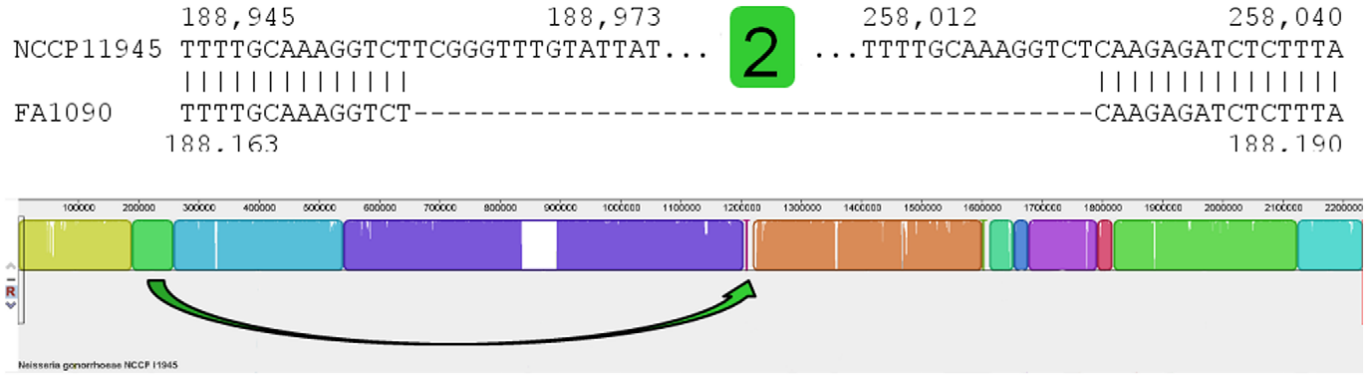
Model for the rearrangement of block 4. Block 4 (purple) is inverted in strain FA1090 relative to the orientation of this region of DNA in strains NCCP11945 (shown) and TCDC-NG08107. At the single base level the regions flanking block 4 from strains NCCP11945 (top) and FA1090 (bottom) align. It is within the flanking regions that homologous recombination is believed to have occurred, between copies of the ∼650 bp Spencer-Smith Repeat Enclosed Elements that flank block 4 (Table 2, Table S7), resulting in the inversion observed (purple arrow; Figure 1).

The NGOФ1 sequence of strains NCCP11945 and TCDC-NG08107 is fragmented into two segments relative to the NGOФ1 annotations of strain FA1090 by Piekarowicz *et al.* [25] (Table S4). The first segment is in the same relative location as strain FA1090 and the second segment is 663 kb away in an inverted orientation, due to the inversion of block 4 (Figures 1 and 3).

**Table 2:** Locations of the Spencer-Smith Repeat Enclosed Elements in *N. gonorrhoeae*.

	NCCP11945			TCDC-NG08107			FA1090		
	Position*	Length	CDSs†	Position*	Length	CDSs†	Position*	Length	CDSs†
SSREE1	540,223–540,873	651 bp	NGK_0658 & NGK_0659	518,752–519,404	653 bp	NGTW0519	468,565–469,216	652 bp	NGO0486 & NGO0487
SSREE2	1,204,392–1,205,041	650 bp	NGK_1449 & NGK_1450	1,119,783–1,120,435	653 bp	NGTW1137	1,065,670–1,066,321	652 bp	NGO1108 & NGO1109
SSREE3	1,605,648–1,606,297	650 bp	NGK_1941 & NGK_1942	1,508,493–1,509,145	653 bp	NGTW1542	1,597,177–1,597,828	652 bp	NGO1637 & NGO1638

* Genomic base positions from the genome sequences.

† Coding sequences annotated between the inverted repeat sequences.

**Figure 4:**
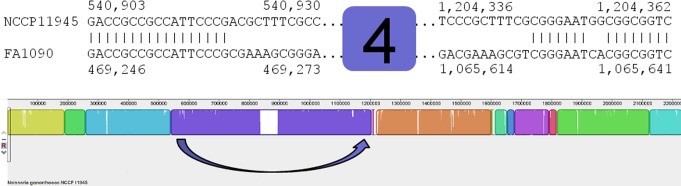
Model for the rearrangements of blocks 5, 7, 8, and 9. All genome sequences investigated have NGOФ3 disrupted by the insertion of NGOФ9, which appears to have been mediated by ISNgo2 elements (Tables S1 and S13). This is schematically illustrated here (Ф9), with base positions of NGOФ9 indicated as in Table S13 for both strains FA1090 (top) and NCCP11945 (bottom). In strains NCCP11945 and TCDC-NG08107, this region has had three further insertions (blue arrow): insertion of block 9 (Figure 1), containing NGOФ6 and a rRNA locus; insertion of block 8 (Figure 1), containing NGOФ8 and a section of genomic DNA; and insertion of block 7 (Figure 1). The relative location of blocks 7, 8, and 9 are schematically illustrated here with base positions from strain NCCP11945. Each rearrangement appears to have occurred separately, mediated by ISNgo2 elements (Tables S1, S10, and S12) and to have displaced block 5 (pink arrow). Note the alignment of homologous sequence flanking the locations of block 5 and blocks 7, 8, and 9, which has been zoomed to the single base level. In addition to the movements of these blocks of sequence, deletion events are also noted within NGOФ3 (Table S6).

As is seen in NGOФ1, the NGOФ2 sequences of strains NCCP11945 and TCDC-NG08107 are fragmented compared to the Piekarowicz *et al.* [25] annotations of this prophage in strain FA1090 (Table S5). The NGOФ2 sequence occurs in four sections in strains NCCP11945 and TCDC-NG08107 (Figure 1). Both NGOФ1 and NGOФ2 have been affected by the inversion of block 4 (Figure 3). Further rearrangements in NGOФ2 involve block 7 and fragmentation by block 5 (Figures 1 and 4).

An ∼600 kb inversion of block 4 has occurred in *N. gonorrhoeae* strains NCCP11945 and TCDC-NG08107, compared to the same block in *N. gonorrhoeae* strain FA1090 (Figures 1 and 3). This inversion has generated the relative location difference of the prophages within the block, NGOФ4 and NGOФ5, in strains NCCP11945 and TCDC-NG08107 compared to strain FA1090 (Figure 1). Directly 5′ of the breakpoints of the inversion of block 4 (Figure 1) are two similar inverted repeats enclosed elements (Table 2). These belong to the sequences of NGOФ1 and NGOФ2, and largely explain the fragmentation and exchange of DNA seen between these two prophage (Figure 1; Tables S4 and S5). These two repeat enclosed elements and a third highly similar element within NGOФ3 (Figure 1; Table 2) are described for the first time here and have been named Spencer-Smith Repeat Enclosed Elements (SSREE).

The three elements, SSREE1 (within NGOФ1), SSREE2 (within NGOФ2), and SSREE3 (within NGOФ3), are similarly sized with a base range of 650 to 653 bp (Table 2) and have near identical inverted repeats of 19 bases: 5′-CGTTTCAGACGGCATCGGG//CCCGATGCCGCCTGAAACG-3′. Pairwise alignment of each SSREE between strains shows >98% similarity (Table S7).

SSREE1 and SSREE2 have remained in the same relative positions in all three strains despite the inversion of block 4 (Figure 1). It therefore appears that the inverted repeats of these SSREE’s have allowed the inversion to occur by providing homologous sequences within the inverted repeats located in NGOФ1 and NGOФ2. These can act as substrates for homologous recombination, facilitating the inverted section reintegrating in the opposite orientation.

### Rearrangements and Deletions within and between NGOФ2 and NGOФ3

Rearrangements have also occurred within the region immediately 3′ of SSREE2. A section of NGOФ2 DNA in *N. gonorrhoeae* strain FA1090 (NGO1110-NGO1111) is absent from the strains NCCP11945 and TCDC-NNG08107 genome sequences and the NGOФ2 DNA sequence immediately 3′ of this is now present within the confines of NGO3Ф (Figures 1 and 4). The sequence of NGOФ3 is found in two segments in *N. gonorrhoeae* strain FA1090 [25], being divided by the insertion of NGOФ9 (Figure 4). The 5′ end of NGOФ3 is further fragmented in strains NCCP11945 and TCDC-NG08107 (Figure 1; Table S6) through the rearrangements of blocks 5 and 7 (Figure 4). The 3′ end of NGOФ3 is highly conserved in all three strains. A region of NGOФ3 DNA making up block 5 is present within the sequence of NGOФ2 in *N. gonorrhoeae* strain NCCP11945 (Figure 4), although not in strain TCDC-NG08107.

### Rearrangements of the ssDNA Prophage

The sequences of NGOФ6 and NGOФ8 (Tables S10 and S12) are located downstream in strains NCCP11945 and TCDC-NG08107 relative to their positions annotated in strain FA1090 (Figure 1) [24], [25] along with two segments of genomic DNA, placing NGOФ6 in block 9 and NGOФ8 in block 8 (Figure 4). Their relative locations to one another are also different, with the NGOФ6-containing block 9 5′ of the NGOФ8-containing block 8 in strain FA1090 and 3′ of block 8 in strains NCCP11945 and TCDC-NG08107. This strain NCCP11945/TCDC-NG08107 configuration places blocks 8 and 9 between the 5′ end of NGOФ3 and NGOФ9 (Figures 1 and 4). It is apparent from comparison of relative prophage locations, and the CDSs flanking these loci, that mobilization and re-integration has occurred in strains NCCP11945 and TCDC-NG08107. Blocks 9 and 8 are both flanked by ISNgo2 elements (Tables S1, S10, and S12).

**Figure 5:**
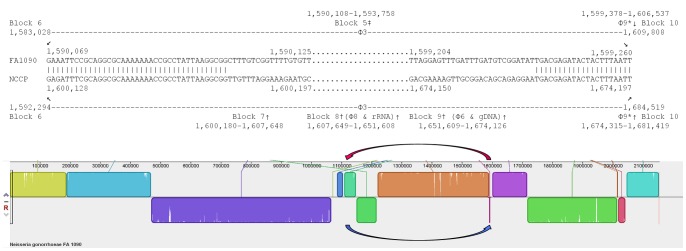
Model for the rearrangement of block 2. Block 2 (green) is present between block 1 (yellow) and block 3 (light blue) in *N. gonorrhoeae* strains NCCP11945 (shown) and TCDC-NG08107. In our model, this region of DNA has been deleted from this location in strain FA1090, making blocks 1 and 3 adjacent (Figure 1). This is shown at the single base level through alignment of sequence to the left and right of the block 2 sequence in strain NCCP11945 (top) with the contiguous sequence of blocks 1 and 3 in strain FA1090 (bottom). The block 2 region of DNA is located elsewhere in the strain FA1090 genome in an inverted orientation (Figure 1). Excision appears to have been via flanking IS1106 elements (Table S3), followed by ISNgo2 element mediated integration using the native ISNgo2 element in block 2 (green arrow; Table S1).

Block 2 is also flanked by ISNgo2 elements in strain FA1090 (Figure 1), however no prophage is present in this genomic segment (Tables S1, S2, S3). In strain FA1090, block 2 starts immediately 3′ of block 8 and finishes immediately 5′ of the ISNgo2 element of NGOФ7 (Figure 1; Tables S1 and S11). In strains NCCP11945 and TCDC-NG08107, block 2 is inverted and much further upstream (Figure 5). An ISNgo2 element is present at the 5′ end of the transposed block 2, yet due to the inversion this is at the opposite end than would be expected. Block 2 is also flanked by a pair of IS1106 elements in strains NCCP11945 and TCDC-NG08107, although there is no trace of IS1106 sequence 5′ of NGOΦ7 in strain FA1090.

### Insertion Sequences

Fifteen IS1016 elements are annotated and a further six are found here in *N. gonorrhoeae* strain FA1090 (Table S2). Two additional sequences are present in *N. gonorrhoeae* strain NCCP11945, in addition to those in strain FA1090. In all cases the IS1016 are within blocks and are not associated with breakpoints in chromosomal synteny. Analysis of features flanking IS1016 shows that all IS1016 elements present in both *N.* *gonorrhoeae* strains are flanked by the same features, so no mobility is apparent. Previously reported genome-wide rearrangements mediated by IS1016 when comparing *N. gonorrhoeae* strain FA1090 to *N. meningitidis* strain Z2491 [22], would appear to either be a rearrangement in strain Z2491 or one that occurred prior to the speciation event that generated *N. gonorrhoeae*.

The complete genome sequences of *N. gonorrhoeae* strains NCCP11945 and TCDC-NG08107 show the presence of six and five IS1106 elements, respectively (Table S3). In addition, each strain also has a putative copy of IS1106A3 (Table S3). In comparison, NCBI MegaBLAST searches using the same relative positions of the annotated IS1106 elements in strains NCCP11945 and TCDC-NG08107 as templates, has revealed four of seven IS1106 elements to be fully intact and present in strain FA1090 in the same relative locations (Table S3). A further two IS1106 elements with 99% similarity were also identified in strain FA1090 (Table S3). There are two IS1106 elements associated with the breakpoints between blocks 1 and 2 and blocks 2 and 3. In strains NCCP11945 and TCDC-NG08107, there is an IS1106 element at the 3' end of block 1 (Figure 1). This element is not present in strain FA1090, where block 2 is in an inverted orientation ∼1 Mb downstream. All three genome sequences have an IS1106 within 200 bp of the 5′ end of block 3 (Figure 1).

In strain NCCP11945, the IS1106 containing NGK_1327 is located around 20 kb downstream of its location in strain TCDC_NG08107 (CDS NGTW08_1054). Analysis of the flanking regions shows that the 5′ and 3′ regions have changed, confirming this transposition event has occurred.

### CREE-mediated Inversion

As reported previously [18], the inversion of blocks 11 to 14 appears to have been mediated by CREE. A single CREE in strain FA1090 at positions 1,715,901 to 1,712,641, spans the breakpoint between blocks 10 and 14 (Figure 1). This sequence aligns with two separate CREE in strain NCCP11945, one at the end of block 10 and one at the end of block 14 (Figure 1), as previously reported [18]. It has been proposed previously that blocks 11 to 14 inverted together by a CREE-mediated mechanism [18]. Further rearrangements within, resulting in blocks 12 and 13 can be attributed to the pilin sequences and rearrangements between them, including the deletion of block 12 in strain TCDC-NG08107, by mechanisms previously investigated in other strains [19], [35], [36].

## Discussion

The largest syntenic difference between the three gonococcal genome sequences from strains NCCP11945, TCDC-NG08107, and FA1090 is the inversion of block 4 (Figures 1 and 3). It would appear that the SSREE inverted repeats identified here have a role in this inversion, but their mechanism of action is unknown. At each end there are IS1016 elements located at around 30 kb and 100 kb of the 5′ and 3′ breakpoints in each strain, which may play a role. The central region of SSREE’s contains one to two predicted CDSs, but these do not appear to encode a transposase. In addition, the positions of the SSREE have not changed, as evidenced by the differences in core regions between SSREE1 and SSREE2, so they do not appear to be mobile. In some ways, the SSREE are similar to the Correia Repeat Enclosed Elements (CREE), which also have inverted repeats and also do not possess a transposase gene. CREE, however, are thought to be mobile and to have contributed to the next largest inversion of blocks 11 to 14 [18], whereas there is no evidence of SSREE elements being mobile. Rather they are located at breakpoints and are possibly the means by which this large chromosomal inversion of block 4 has been mediated.

Overall it is not clear where the sequences of NGOФ2 and NGOФ3 become intertwined and in which of the lysogenic phage the respective CDSs originated. A high degree of similarity between the sequences of lysogenic phage NGOФ1, NGOФ2, and NGOФ3 has been previously outlined [25]. Some of the exchanged areas also contain only prophage CDSs encoding putative phage associated proteins with unknown function, however the additional block of CDSs 3′ of the NGOФ2 breakpoint that is present only in *N. gonorrhoeae* strains NCCP11945 and TCDC-NG08107 contains some genes of function. This entire block of CDSs may have originated as part of NGOФ2, and the presence of the highly similar DNA replication protein already found in NGOФ3 would further suggest this. It does, however, seem apparent that the rearrangements seen here are largely associated with the SSREE element. NGOФ3 has been shown to be incomplete in strain FA1090 [25], and it would appear that the NGOФ3 sequences of strains NCCP11945 and TCDC-NG08107 show successive and continued degradation, respectively.

The fragmentation seen between lysogenic phage NGOФ2 and NGOФ3, the large inversion between NGOФ1 and NGOФ2, and the relatively high similarity seen in a significant proportion of all three of these prophage, raises questions on their native configuration and gene compliment. It is apparent that the inversion occurred between SSREE1 and SSREE2. Also of interest is the orientation of these phage in comparison to each other. The majority of the NGOФ1 CDSs 5′ of the inversion are homologous to the majority of the NGOФ2 CDSs 3′ of the inversion. In fact it has previously been stated by Piekarowicz *et al.* [25] that the phage are ordered opposing to each other. The inversion therefore occurs between the largely dissimilar regions of the two prophage and therefore no CDSs appear duplicated or deleted by this inversion. The sequence of NGOФ2 also appears more complete in *N. gonorrhoeae* strains NCCP11945 and TCDC-NG08107, which is evident in the block of additional CDSs not present in strain FA1090. The areas of NGOФ2 and NGOФ3 in strains TCDC-NG08107 and NCCP11945 that appear to be exchanged compared to strain FA1090 may have been native in strains TCDC-NG08107 and NCCP11945. There is also the exchange of several CDSs between NGOФ2 and NGOФ3, which seems equally likely to occur in the NCCP11945/TCDC-NG08107 configuration as the FA1090 configuration.

An almost full gene complement is seen in all filamentous prophage sequences (NGOФ6, NGOФ7, NGOФ8, and NGOФ9) between strains and copies. This, combined with the change in location of two of these prophage (NGOФ6 and NGOФ8), suggests they are still functional. What is unexpected is the mechanism by which they appear to have changed location. It has previously been suggested that ISNgo2 elements of filamentous prophage mediate integration of the phage into the *N. gonorrhoeae* genome [22]. However, this theory states that a single copy of ISNgo2 is used for prophage mobilization and integration, with the mobilized phage forming a circular, plasmid-like intermediate.

This study suggests that the filamentous prophage may mobilize and integrate in much the same way as composite transposons, therefore requiring two copies of an ISNgo element. Only the ISNgo2 at the 5′ end is taken with the transposing block of DNA and the consensus sequence of another ISNgo2 element found elsewhere in the genome is required for it to reintegrate. It appears that in the case of NGOФ6 and NGOФ8, the ISNgo2 element associated with the mobilized prophage works in tandem with a downstream ISNgo2 copy, which remains in its original location. It may be that the native state of filamentous prophage is to have a copy of ISNgo2 and ISNgo3 flanking the phage sequence (as seen with NGOФ9), this would allow transposon-like movements within the genome to occur without large sections of genomic DNA transposing with them. Further evidence of this is seen in strains NCCP11945 and TCDC-NG08107 where a second and third ISNgo3 element is present, respectively. It should be noted, however, that none of the ISNgo3 elements are found in differing locations between strains.

It is tempting to speculate that the sequences of NGOФ6 and NG0Ф8, along with the genomic DNA that has transposed with them, originated in the position that they are found in *N. gonorrhoeae* strain FA1090. These prophage and their associated genomic DNA are found interrupting the genome of lysogenic prophage NGOФ3 in *N. gonorrhoeae* strains NCCP11945 and TCDC-NG08107. It may be that the 5′ ISNgo2 element of NGOФ9 already inserted within the NGOФ3 genome provides a 3′ ISNgo2 element for the mobilized segments to insert at this loci. NGOФ9 is present within NGOФ3 in all strains analyzed and would have inserted there after the insertion of NGOФ3 into the gonococcal genome (Figure 4).

**Figure 6:**
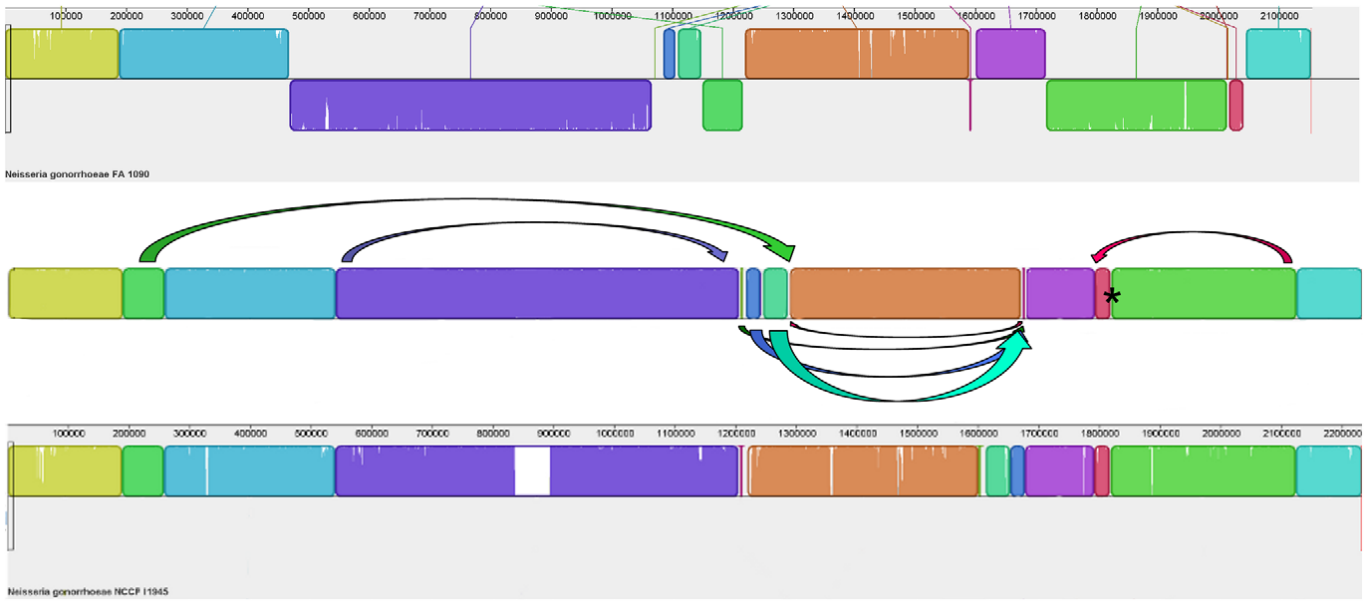
Model for the chromosomal rearrangements observed in *N. gonorrhoeae* strains NCCP11945 and FA1090. Alignments of the genome sequence data shows that the organisation of the genomes of strains NCCP11945 and FA1090 are different (Figure 1). Analysis of this data reveals that the most likely original order of the genome is that shown in the centre of this figure from left to right as blocks 1 (yellow), 2 (green), 3 (light blue), 4 (purple), 7 (lime), 9 (blue), 8 (teal), 6 (orange), 5 (pink), 10 (violet), 11 (red), 12 (too small to be visible), 13 (too small to be visible), and 14 (bright green), circularising back to block 1 (aqua). In strain FA1090 (top), IS1106-mediated homologous recombination and ISNgo2 mediated reintegration have caused a translocation and inversion of block 2 (green arrow), SSREE-mediated homologous recombination has inverted block 4 (purple arrow), and CREE-mediated homologous recombination has inverted blocks 11–14 together (red arrow). In strain NCCP11945 (bottom), ISNgo2 mediated excision and reintegration have caused the translocation of block 7 (lime arrow), block 9 (blue arrow), and block 8 (teal arrow), and the displacement of block 5 (pink arrow).

In *N. gonorrhoeae* strains NCCP11945 and TCDC-NG08107, block 9 containing NGOФ6 and block 8 containing NGOФ8 have not only transposed between the 5′ end of NGOФ3 and NGOФ9, but have also inserted in the opposite order, with block 8 preceding block 9 (Figure 6). This may have occurred in two steps where one block transposed 5′ of NGOФ9 and then the second block inserted also 5′ of NGOФ9 or 5′ of the first block to transpose (Figure 4). Both cases would provide a 3′ ISNgo2 copy and the consensus sequence necessary for this reintegration. It seems less likely that blocks 8 and 9 transposed together and then reversed order due to a second transposition event.

It is possible that block 2 transposed from its location in strain FA1090 to its location upstream in gonococcal strains NCCP11945 and TCDC-NGO8107 after the transposition of the blocks 8 and 9. As stated previously, two copies of ISNgo2 are required for transposition. Blocks 8 and 9 would take with them the 5′ ISNgo2 during the transposition seen in strains NCCP11945 and TCDC-NG08107. This would leave Block 2 with a 5′ and 3′ copy of ISNgo2. What is unusual is that block 2 is found inverted in strains NCCP11945 and TCDC-NG08107, yet the ISNgo2 is found at what was originally the 3′ end of the block. This raises doubt as to whether ISNgo2 mediated this rearrangement seen in strains NCCP11945 and TCDC-NG08107. If block 2 originated where it is in strain FA1090, its transposition might be explained by the IS1106 elements that flank block 2 in strains NCCP11945 and TCDC-NG08107, however no trace of them is found 5′ of NGOФ7 in any of the three *N. gonorrhoeae* strains. If, however, block 2 originated where it is in strains NCCP11945 and TCDC-NG08107 then it is possible that the block 2 sequence was excised from the chromosome due to homologous recombination between the flanking IS1106 elements and was then able to reintegrate 3′ of block 8 through its own ISNgo2 and the one resident at the end of block 8 in strain FA1090 (Figure 6). This explanation can account for the presence of an ISNgo2 only at the 3′ end of block 2 in strains NCCP11945 and TCDC-NG08107, whereas the element is found at the 5′ of blocks where it has mediated rearrangement independently of IS1106. Activity of the IS1106 containing NGK_1327 suggests that other IS1106 elements in the genome could be active and capable of mobilization. Block 2 could have been excised from the genome via an IS-mediated mechanism, taking all of block 2, flanked by IS1106 elements in a composite transposon-like manner. This alternative mechanism, rather than through homologous recombination between the IS1106 copies, would generate the same end result and would be supported by our model (Figure 6).

We therefore present a model where the original organization of the chromosome ordered the blocks 1, 2, 3, 4, 7, 9, 8, 6, 5, 10, 11, 12, 13, and 14 (Figure 6). To generate the block order seen in *N.* *gonorrhoeae* strain FA1090, block 2 was excised by homologous recombination between flanking IS1106 elements and reintegrated by virtue of the ISNgo2 within block 2 and that at the end of block 8 (Table S1). In addition, homologous recombination between the SSREE repeats flanking block 4 (Table 2) and the CREE repeats flanking blocks 11 to 14 [18] caused the inversion of these blocks in strain FA1090 (Figure 6). The chromosomal organization seen in *N. gonorrhoeae* strains NCCP11945 and TCDC-NG08107 has arisen through the movement of ISNgo2-containing regions, moving blocks of sequence 7, 8, and 9. This displaces block 5, which reintegrates between blocks 4 and 6 in strain NCCP11945 (Figure 6) and is deleted in strain TCDC-NG08107. The remaining minor differences in chromosomal arrangement are due to recombination within the pilin gene loci in blocks 12 and 13 (Figure 6), with the former having been deleted in strain TCDC-NG08107. By this model, the changes to the strain FA1090 genome are largely mediated by recombination between IS-like repeats, while the changes in strains NCCP11945 and TCDC-NG08107 are due to ISNgo2 elements. This may indicate that prophage activation has played a role in the location of the prophage, prophage-associated sequences, and genomic sequences associated with ISNgo2 in strains NCCP11945 and TCDC-NG08107.

### Conclusions

The majority of the breakpoints in chromosomal synteny (Figure 1; Table 1) seen when comparing the genome sequence of *N. gonorrhoeae* strain NCCP11945 to those of *N. gonorrhoeae* strains TCDC-NG08107 and FA1090, appear to have been the result of prophage/IS element mobilization and reintegration and associated with the IS element-like CREE [18] and SSREE sequences; the remaining rearrangements appear to have arisen as a result of recombination between pilin genes (Figure 6). The inversion of block 4 (Figure 3), and the translocations of blocks 5 and 7 (Figure 4), result in rearrangements between the three lysogenic prophage: NGOФ1; NGOФ2; and NGOФ3 (Figure 1). The translocations of blocks 8 and 9 (Figures 1 and 4), and the translocation and inversion of block 2 (Figures 1 and 5), are attributed to the ISNgo2 elements of filamentous prophage NG0Ф6, NGOФ8, and additional ISNgo2 elements within the genomes of filamentous prophage NGOФ7, NGOФ9, and those ISNgo2 elements not affiliated with prophage. Therefore, activation of prophage, IS, and IS-like element mobilization is key to the chromosomal rearrangements seen when comparing gonococcal genome sequences.

## Supporting Information

Table S1 Locations of ISNgo2 and ISNgo3 elements in *N.* *gonorrhoeae* strains FA1090, NCCP11945 and TCDC-NG08107.
(XLS)

Table S2 Locations of IS1016 elements in *N. gonorrhoeae* strains FA1090 and NCCP11945.
(XLS)

Table S3 Locations of IS1106 in *N. gonorrhoeae* strains FA1090, NCCP11945 and TCDC-NG08107.
(XLS)

Table S4 Lysogenic dsDNA prophage NGOФ1 in *N. gonorrhoeae* strains FA1090, NCCP11945 and TCDC-NG08107.
(XLS)

Table S5 Lysogenic dsDNA prophage NGOФ2 in *N. gonorrhoeae* strains FA1090, NCCP11945 and TCDC-NG08107.
(XLS)

Table S6 Lysogenic dsDNA prophage NGOФ3 in *N. gonorrhoeae* strains FA1090, NCCP11945 and TCDC-NG08107.
(XLS)

Table S7 Percent similarity of SSREEs in *N. gonorrhoeae* strains.
(XLS)

Table S8 Lysogenic dsDNA prophage NGOФ4 in *N. gonorrhoeae* strains FA1090, NCCP11945 and TCDC-NG08107.
(XLS)

Table S9 Lysogenic dsDNA prophage NGOФ5 in *N. gonorrhoeae* strains FA1090, NCCP11945 and TCDC-NG08107.
(XLS)

Table S10 ssDNA prophage NGOФ6 in *N. gonorrhoeae* strains FA1090, NCCP11945 and TCDC-NG08107.
(XLS)

Table S11 ssDNA prophage NGOФ7 in *N. gonorrhoeae* strains FA1090, NCCP11945 and TCDC-NG08107.
(XLS)

Table S12 ssDNA prophage NGOФ8 in *N. gonorrhoeae* strains FA1090, NCCP11945 and TCDC-NG08107.
(XLS)

Table S13 ssDNA prophage NGOФ9 in *N. gonorrhoeae* strains FA1090, NCCP11945 and TCDC-NG08107.
(XLS)
